# How Confident Can We Be in Modelling Female Swimming Performance in Adolescence?

**DOI:** 10.3390/sports4010016

**Published:** 2016-03-03

**Authors:** Shilo J. Dormehl, Samuel J. Robertson, Craig A. Williams

**Affiliations:** 1 University of Exeter, Children’s Health and Exercise Research Centre, College of Life and Environmental Sciences, St Luke’s Campus, Heavitree Road, Exeter EX1 2LU, UK; sjd223@exeter.ac.uk; 2 Victoria University, Institute of Sport, Exercise & Active Living, Melbourne 3011, Australia; Sam.Robertson@vu.edu.au

**Keywords:** puberty, tracking, talent-development, sub-elite, competition

## Abstract

The purpose of this research was to determine the expected progression of adolescent female swimming performances using a longitudinal approach. The performances of 514 female swimmers (12–19 year olds) who participated in one or more FINA-regulated annual international schools’ swimming championships over an eight-year period were analysed. Quadratic functions for each of the seven individual events (50, 100, 200 m freestyle, 100 m backstroke, breaststroke, butterfly, 200 m individual medley) were determined using mixed linear models. The predicted threshold of peak performance ranged from 16.8 ± 0.2 (200 m individual medley) to 20.6 ± 0.1 (100 m butterfly) years of age, preceded by gradual rates of improvement (mean rate of 1.6% per year). However, following cross validation, only three events (100 m backstroke, 200 m individual medley and 200 m freestyle) produced reliable models. Identifying the factors that contribute to the progression of female performance in this transitory period of life remains challenging, not least since the onset of puberty is likely to have occurred prior to reaching 12 years of age, the minimum competition age for this championship.

## 1. Introduction

Based on the increasing pressure for nations to develop talented athletes and win medals at the highest level, many sporting bodies have directed strategies and resources to increasing performance levels in all sports; swimming is no exception [1,2,3]. Trying to separate the performance gains that are made by athletes due to training as opposed to natural growth and development has been one of the most important challenges to overcome. Malina [4] highlighted the need for longitudinal studies to better understand how and when athletes’ performances progressed. There have been a number of approaches to predictive modelling in a variety of different sports, including physiological, mathematical or probability strategies [5]. However, these authors suggest that until all factors such as biomechanical, physiological and psychological parameters that influence human performance are fully understood and accounted for, modelling will continue to lack sufficient accuracy to meaningfully predict future performance. Nevertheless, numerous studies have considered how changes in physical, physiological and biomechanical parameters affect performance during adolescence [6,7].

To date, research exploring the development of youth swimmers during adolescence has focussed mainly on male subjects [8,9,10,11] with comparatively fewer targeting solely young females [7,12]. In one of the few studies on young female swimmers, Lätt, Jürimäe, Haljaste, Cicchella, Purge and Jürimäe [7] found that development of biomechanical factors such as velocity, stroke length, stroke rate and in particular stroke index, rather than bioenergetics, contributed more to improved performance times in the 400 m freestyle event.

The performance gap between adult males and females in swimming has reportedly been stable at 8.9% since 1979 [13]. Despite the negligible differences in swimming performance between the sexes before puberty, from age 12 years onwards the performance gap appears to increase [14]. Indeed, it is the greater stroke-specific power of males compared with females that is purported to be a key contributing factor to this difference [15,16]. However, it has been proposed that since females mature physically earlier than males, they are better equipped to compete equitably with older females after reaching the age of 15 years [14]. From a physical standpoint, males can only start competing with an equal chance of success against mature males from the age of 17 years [14].

Baxter-Jones [17] questioned the age at which athletes should formally start competing and this debate remains as relevant today. In contradiction to competition entry requirements, the Amateur Swimming Association’s (ASA) “The Swimmer Pathway” [18] advocated that only 15 year old female swimmers should consider racing at the “training to compete” stage of the Long Term Athlete Development model [19]. However, Grange and Gordon [20] indicated that the youngest competition age was 9 years and the distances over which these younger swimmers competed continued to change, with no distinction being made between sexes [21]. Furthermore, the latest version of the ASA handbook does not make any reference to race distances for these younger swimmers [22]. Despite this, Light, Harvey and Memmert [3] found that, given the appropriate setting, club swimmers drawn from France, Germany and Australia (mean age of 10.39 ± 1.07 years), were in fact demonstrating early specialisation and were not averse to competing at an early age. The findings of Barynina and Vaitsekhovskii [23] suggested that young swimmers would benefit from later specialisation within the sport (after the age of 12 years) and less training before reaching the age of 11 years. These findings add support to the sampling approach to sport advocated by the Development Model of Sports Participation [24]. However, Erlandson, Sherar, Mirwald, Maffulli and Baxter-Jones [12] found the development process of young female elite athletes did not appear to be adversely affected by intensive participation in sports, including swimming. The multitude of conflicting ideas regarding the minimum age for specialisation and/or competition suggested by various research groups, sporting bodies and development models confirms that, as yet, there is no definitive conclusion to this debate.

Longitudinal studies have the potential to help coaches gain perspective on the success of young athletes and enables them to give sound career advice [8]. A longitudinal study by Sokolovas [25] was one of the first to draw attention to the value of tracking elite swimmers retrospectively through their careers. With recent improvements in statistical methods, Allen *et al.* [26] and Dormehl *et al.* [27] have extended this concept by creating mixed linear models of elite-level, and sub-elite adolescent male swimmers respectively.

Since there are many challenges associated with constructing accurate models of human performance, besides the performance of young female sub-elite swimmers, it is unsurprising that no quantifiable baseline model currently exists. While it is tempting to create an all-encompassing model of swimming as a single sport, it is of more value to coaches and swimmers to acknowledge the individual specialisms within this multi-disciplinary sport. The aim of the present study was therefore to create the first models of the performance progression of sub-elite adolescent female swimmers for common strokes and distances. Identifying the threshold ages of peak performance in adolescent female swimmers could provide coaches and sporting associations with some potentially useful benchmarking tools to identify talent, and possibly provide evidence to determine realistic qualifying times as well as a justifiable minimum competition age for females.

## 2. Methods

Performance times for all female entrants (*n* = 514, aged between 12–19 years) who competed in one of seven individual events (Table 1) were extracted from the official results of an annual schools’ swimming championships from 2006 to 2013. The 13 competing schools were American, British and International schools, predominantly located in Western Europe. Team sizes were limited and the competition rules limited swimmers to a maximum of three individual events per championship. The data were in the public domain and downloaded from the relevant tournament websites. All swimmers were assigned individual identity codes to ensure anonymity. The study was approved by the institutional ethics committee and conformed to the recommendations of the Declaration of Helsinki. The single best performances in each of the seven events entered (in either the heats or the finals) over the 8-year analysis period are described in Table 1. The swimmers’ ages at the time of each competition were also obtained.

### 2.1. Statistical Analysis

The raw datasets for all performances in each of the seven events were tested for normality using the Shapiro–Francia test [28] in STATA ver. 13 (StataCorp. 2013. *Stata Statistical Software: Release 13*. College Station, TX, USA: StataCorp LP). The trajectories of the curves showing the progression in performance during maturation were analysed using mixed or multi-level modelling (MLM) in STATA. Time was zero centred at 12 years of age, using an unstructured covariance approach. The fit of the models in fixed and random effects were compared with maximum likelihoods, using a hierarchical method. The final models were quadratic functions for fixed effects (*y* = a*x*^2^ + b*x* + c). The fixed effects of time represented polynomial changes of the population with age and the random effects reflected individual deviations from the sample mean trajectory. Inter-class correlation coefficients were calculated and R^2^ values determined in order to measure the difference between and within person variability and effect size respectively.

### 2.2. Evaluation of Models

The datasets for certain events had non-normal distributions. As a result, to validate the proposed models, cross-validations were performed whereby the datasets were randomly split into 1/3 and 2/3 sub-groups. Cross-validation of models is highly recommended under such circumstances in order to determine the generalisability of the findings [29].

The percentage rate of improvement was determined through differentiation of the quadratic functions for each event separately, as $y=\left(\frac{2a}{c}\;\times \;100\right)x+\left(\frac{b}{c}\;\times \;100\right)$, where *y* = percent change in performance time and *x* + 12 = age, in years. The threshold age of peak performance was calculated as the axis of symmetry of the quadratic function *i.e.*, $\frac{-b}{2a}$.

## 3. Results

Many of the probability values for the coefficients of the functions were greater than 0.05 (Table 2), resulting in reduced confidence in those models. This included the full model of the fixed quadratic for the 100 m butterfly and at least one of the cross-validation models for the 50 and 100 m freestyle in addition to the 100 m backstroke and breaststroke. Cross validation confirmed that the full models for the 200 m freestyle and the 100 m backstroke events fit the data well in comparison to those for the other events. In the remaining five events however, at least one coefficient of the cross-validation models fell just outside of the standard error (SE) of the full model, but all fell within the 95% confidence interval (C.I.) of the full model. Of all the models, the 100 m freestyle event had the poorest fit.

**Figure 1:**
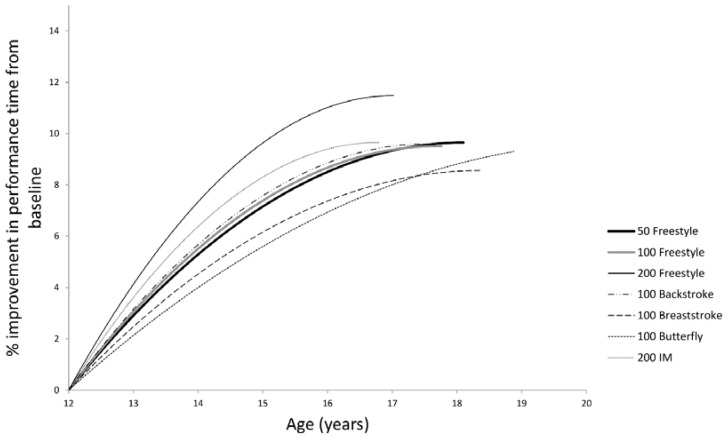
Quadratic functions of the progression in performance for each of the seven events modelled for females from the baseline age of 12 years to the threshold age of peak performance.

The models indicate that female swimmers are likely to reach their threshold of peak performance earliest in the 200 m individual medley (16.8 years) and latest in the 100 m butterfly, the latter of which was predicted to occur beyond the age range of the dataset (Figure 1 and Table 3). The slowest rate of improvement between the ages of 12 and 16.8 years was observed in 100 m butterfly swimmers, whereas the greatest rate of improvement (over the same age range) was predicted to occur in the 200 m freestyle event. For the modelled improvement rates from 12 years through to the threshold age, 200 m freestyle swimmers remain the fastest improving, while breaststroke swimmers replace butterfly swimmers as the slowest to improve (Table 3).

## 4. Discussion

The aim of the study was to model the performance of female swimmers in all strokes between the ages of 12 and 19 years. However, only the 200 m freestyle, the 100 m backstroke and, to a lesser extent, the 200 m individual medley events produced functions that can be interpreted with any confidence (Table 2).

Although Kojima, Jamison and Stager [14] did not aim to determine a peak age—they predicted that females could already start competing equally with older females from as young as 15 years of age. In contrast, the quadratic functions of this study indicated thresholds of peak performance occurred later, i.e. from the age of 16.8 years (Table 3). A possible reason for this apparent discrepancy is that our dataset only included females from the age of 12 years (Figure 1), as this was the minimum entry age for the particular competition studied, while in the Kojima study there were swimmers as young as 7 years of age [14]. The unexpectedly late age of predicted peak performance for swimmers competing in the 100 m butterfly (20.6 years), was largely due to the shallow gradients (approx. 1.1% per year) of modelled improvement for this event. According to Malina, *et al.* [30], puberty begins at approximately 8 to 10 years of age for females and the mean age of menarche has been reported as 12.9 years [31]. It is therefore possible that the majority of females in this study may already have experienced meaningful gains in performance due to maturational development prior to competing in these events. 

The threshold age of peak performance for the sub-elite female swimmers in this study were on average only 0.7 years younger than their male counterparts at the same championships [27], even though females are expected to mature approximately 2 years earlier [30]. This finding supports the authors’ concerns about combining data on all strokes and distances into one single model, as the relatively late predicted age of peak performance in the butterfly will undoubtedly have contributed to the higher mean threshold age calculated for the females in this study. However, the relative rate of improvement for adolescent female swimmers is confounded by numerous additional factors. Since females mature earlier than males, their improvement between the ages 12 and 19 years is likely to be affected less by biological processes and potentially more by external factors, including biomechanical development, psychological and social pressures [32]. While the growth and maturational process to adulthood starts prior to the age of 12 years for females, it has been questioned whether they have sufficient cognitive development to deal with the rigours of high level competition and the concomitant pressures [33], or whether they should be specialising at such a young age [34].

The expected plateau in performance as biological maturation nears its peak, experienced earlier in females than males, is a factor possibly leading to waning interest and commitment to training and potentially higher dropout rates in females [12]. In accordance with the findings of Cornett and Stager [35], who examined the effect of the number of entrants in a 50 yard freestyle event on the level of performance, it is also possible that the lower number of entrants in the older age groups (data not shown) may also have contributed to reduced competitiveness in these groups. Nevertheless, these sub-elite females were predicted to attain their threshold of peak performance 5.1 years earlier than the peak performance age reported for elite-level swimmers in the same events analysed by Allen, Vandenbogaerde and Hopkins [26]. The difference is likely due in part to their study exclusively containing a narrower sample of elite swimmers and, importantly, included performance data that progressed beyond their teenage years.

While the predicted models in this study provide poor fit for many of the events, there is value in examining the comparisons between events. Females reach their threshold of peak performance in longer distance events such as the 200 m individual medley and the 200 m freestyle at a younger age than shorter distance events (Table 3), confirming a phenomenon reported on by Arellano, *et al.* [36] and Allen, Vandenbogaerde and Hopkins [26]. Swimmers competing in the 200 m freestyle event also demonstrated the highest rate of improvement between the ages of 12 and 16.8 years (Figure 1). It is possible that females improve most in the longer distance events due to changes in body composition as a result of puberty. Post-pubertal females are known to have greater buoyancy, which has been suggested to give them an energy efficiency advantage over males [37] and is most noticeable in longer distance events [13,38].

### Practical Applications

Rather than being limited to mere mathematical comparisons of combined threshold times of numerous specialisms within swimming, the value of the individual models developed in this study promotes many potential applications for coaches, swimmers and governing bodies. Swimmers can set realistic targets for the following season and coaches can measure the performance of their adolescent female swimmers against the average expected progressions for each of the events modelled. Furthermore, swimmers who consistently exceed the modelled rates of progression might be considered for talent development or alternatively may be identified as early or late maturers. With further refinements of the models, they could one day also assist governing bodies in the setting of justifiable qualifying times for national and international competitions.

## 5. Conclusions

Despite the poor fit of some of the models generated, the novel analysis of individual events allows for some interesting comparisons to be made. The authors feel that this approach is of more value than a one size fits all model for the sport. The models suggest that females achieve thresholds of peak performance earlier in longer distance events. Use of this particular international schools’ swimming competition provided a consistent minimum age over many consecutive years and consequently ensured high validity of the dataset. However, the slow rate of progression seen in the quadratic functions generated in comparison to those found for the male adolescents by Dormehl, Robertson and Williams [27] indicates that the process of maturation had likely already begun for many of the females in this study. Compared with data for male swimmers [27], confidently identifying the contribution of maturation to performance improvement in females through adolescence remains an elusive goal. Future research should therefore consider collecting longitudinal data on very young swimmers in competition, as these could generate more robust models and higher levels of confidence. Finding a suitable sub-elite competition setting for this may however prove difficult until such time as a consensus is reached on a suitable minimum age of competition, and whether this age should be the same for both males and females. Overcoming these issues could lead to the development of useful benchmarking tools for potential talent identification of sub-elite athletes or the setting of realistic development goals.

## Figures and Tables

**Table 1:** Cumulative number of performances over the 8-year analysis period (between 2006 and 2013) for female swimmers between the ages of 12 and 19 years in each event.

Number of Performances (Years)	50 m Freestyle	100 m Freestyle	200 m Freestyle	100 m Backstroke	100 m Breaststroke	100 m Butterfly	200 m Individual Medley
1*	414	310	233	223	217	135	163
2	167	109	92	83	84	48	64
3	69	42	28	34	33	22	23
4	22	17	10	14	12	8	8
5	7	3	5	5	3	6	2
6	2	0	2	0	1	2	0

Note: The drop in the number of repeat performances was likely to have been caused by a change in event choice, team selection, the transitory nature of scholars at international schools, injury or dropout. * This row of data denotes the total number of swimmers competing in each event, since this table sums the consecutive number of years swum. *i.e.*, the total number of entrants in the 50 m freestyle event was 414, 167 of whom competed for two or more years with 2 of whom went on to swim in this event for 6 consecutive years (the maximum number of years over which any swimmer could compete between age 12 and 19 years).

**Table 2:** Summary of models for all events with cross validation for each of the fixed effects of the quadratic functions.

Predictor	50 m Freestyle		100 m Freestyle		200 m Freestyle		100 m Backstroke		100 m Breaststroke		100 m Fly		200 m Individual Medley	
	Mean	*p*	Mean	*p*	Mean	*p*	Mean	*p*	Mean	*p*	Mean	*p*	Mean	*p*
Fixed Quadratic (a)	0.095	<0.001	0.24	<0.001	0.83	<0.001	0.29	<0.001	0.217	0.012	0.12	0.333	0.83	<0.001
(SE)	(0.02)		(0.06)		(0.13)		(0.07)		(0.09)		(0.12)		(0.19)	
95% C.I.	0.05		0.11		0.25		0.13		0.17		0.24		0.37	
Cross val. 2/3 diff.	−0.03	<0.001	0.10	0.072	0.071	<0.001	−0.05	<0.001	−0.001	0.054	0.048	0.637	0.035	0.001
Cross val. 1/3 diff.	0.03	0.089	−0.11	<0.001	0.045	0.001	0.01	<0.077	−0.03	0.128	−0.09	0.299	−0.26	0.001
Fixed Linear (b)	−1.16	<0.001	−2.73	<0.001	−8.31	<0.001	−3.22	<0.001	−2.77	<0.001	−2.05	0.031	−7.96	<0.001
(SE)	(0.17)		(0.45)		(0.97)		(0.52)		(0.63)		(0.95)		(1.40)	
95% C.I.	0.34		0.87		1.90		1.02		1.24		1.86		2.73	
Cross val. 2/3 diff.	0.22	<0.001	−0.73	<0.001	−0.53	<0.001	0.19	<0.001	0.22	<0.001	−0.54	0.196	−0.202	<0.001
Cross val. 1/3 diff.	−0.15	<0.001	0.72	<0.001	−0.30	<0.001	−0.51	0.001	−0.67	0.027	1.13	0.045	1.26	0.002
Fixed Intercept in seconds (c)	36.69	<0.001	81.66	<0.001	181.21	<0.001	93.06	<0.001	103.25	<0.001	90.59	<0.001	197.62	<0.001
(SE)	(0.32)		(0.92)		(2.1)		(1.10)		(1.19)		(1.79)		(2.86)	
95% C.I.	0.62		1.81		4.11		2.15		2.33		3.51		5.60	
Cross val. 2/3 diff.	−0.27	<0.001	1.29	<0.001	1.03	<0.001	0.59	<0.001	−0.42	<0.001	1.37	<0.001	1.36	<0.001
Cross val. 1/3 diff	−0.12	<0.001	−1.27	<0.001	−0.03	<0.001	−0.68	<0.001	1.30	<0.001	−2.89	<0.001	−2.44	<0.001
ICC	0.90		0.96		0.97		0.97		0.95		0.90		0.97	
χ^2^ df	133.12 [df = 5]		70.41 [df = 7]		93.31 [df = 7]		60.32 [df = 7]		46.47 [df = 7]		26.4 [df = 5]		45.41 [df = 7]	
Total R^2^	0.03		0.05		0.08		0.04		0.02		0.04		0.17	
*n*	414		310		233		223		217		135		163	

Notes: Cross val. diff. is the difference between the cross validation split and the whole sample mean; SE = standard error; C.I. = confidence interval; df = degrees of freedom.

**Table 3:** Descriptors determined for the full models of the seven events.

Predictor	50 m Freestyle	100 m Freestyle	200 m Freestyle	100 m Backstroke	100 m Breaststroke	100 m Fly	200 m Individual Medley
% Rate of improvement (12 year−threshold age)	9.65	9.50	11.48	9.60	8.56	9.66	9.66
% Rate of improvement (from 12 to 16.8 year)	9.21	9.28	11.46	9.43	8.04	7.81	9.66
Threshold age at peak performance (year)	18.1 (0.02)	17.8 (0.06)	17.0 (0.13)	17.6 (0.07)	18.4 (0.09)	20.6 (0.12)	16.8 (0.19)
Performance time (s) at threshold age	33.15 (0.14)	73.81 (0.14)	160.42 (4.87)	84.04 (0.36)	94.42 (0.32)	81.85 (0.11)	178.50 (6.70)

## References

[1] Barreiros A., Côté J., Fonseca A.M. (2014). From early to adult sport success: Analysing athletes' progression in national squads. Eur. J. Sport Sci..

[2] Bergeron M.F., Mountjoy M., Armstrong N., Chia M., Côté J., Emery C.A., Faigenbaum A., Hall G., Kriemler S., Léglise M. (2015). International Olympic committee consensus statement on youth athletic development. Br. J. Sports Med..

[3] Light R.L., Harvey S., Memmert D. (2013). Why children join and stay in sports clubs: Case studies in Australian, French and German swimming clubs. Sport Educ. Soc..

[4] Malina R.M. (1994). Physical-activity and training: Effects on stature and the adolescent growth spurt. Med. Sci. Sports Exerc..

[5] Liu Y., Paul S., Fu F.H. (2012). Accomplishments and compromises in prediction research for world records and best performances in track and field and swimming. Meas. Phys. Educ. Exerc. Sci..

[6] Baxter-Jones A.D.G., Goldstein H., Helms P. (1993). The development of aerobic power in young athletes. J. Appl. Physiol..

[7] Lätt E., Jürimäe J., Haljaste K., Cicchella A., Purge P., Jürimäe T. (2009). Physical development and swimming performance during biological maturation in young female swimmers. Coll. Antropol..

[8] Costa M.J., Marinho D.A., Bragada J.A., Silva A.J., Barbosa T.M. (2011). Stability of elite freestyle performance from childhood to adulthood. J. Sports Sci..

[9] De Mello Vitor F., Böhme M.T.S. (2010). Performance of young male swimmers in the 100-meters front crawl. Pediatr. Exerc. Sci..

[10] Barbosa T.M., Costa M., Marinho D.A., Coelho J., Moreira M., Silva A.J. (2010). Modeling the links between young swimmers' performance: Energetic and biomechanic profiles. Pediatr. Exerc. Sci..

[11] Baxter-Jones A.D.G., Helms P., Maffulli N., Bainespreece J.C., Preece M. (1995). Growth and development of male gymnasts, swimmers, soccer and tennis players: A longitudinal study. Ann. Hum. Biol..

[12] Erlandson M.C., Sherar L.B., Mirwald R.L., Maffulli N., Baxter-Jones A.D.G. (2008). Growth and maturation of adolescent female gymnasts, swimmers, and tennis players. Med. Sci. Sports Exerc..

[13] Thibault V., Guillaume M., Berthelot G., El Helou N., Schaal K., Quinquis L., Nassif H., Tafflet M., Escolano S., Hermine O. (2010). Women and men in sport performance: The gender gap has not evolved since 1983. J. Sports Sci. Med..

[14] Kojima K., Jamison P.L., Stager J.M. (2012). Multi-age-grouping paradigm for young swimmers. J. Sports Sci..

[15] Toussaint H.M., Hollander A.P., Berg C., Vorontsov A., Garrett W.E., Kirkendall D.T. (2000). Biomechanics of swimming. Exercise and Sport Science.

[16] Wells G.D., Schneiderman-Walker J., Plyley M. (2006). Normal physiological characteristics of elite swimmers. Pediatr. Exerc. Sci..

[17] Baxter-Jones A.D.G. (1995). Growth and development of young athletes: Should competition levels be age related?. Sports Med..

[18] Amateur Swimming Association (2003). The Swimmer Pathway: Long Term Athlete Development.

[19] Balyi I., Hamilton A. (2004). Long-Term Athlete Development: Trainability in Childhood and Adolescence—Windows of Opportunity, Optimal Trainability.

[20] Grange J., Gordon R. (2004). Success is Long Term: Long Term Athlete Development Related to the Journey through Swimming.

[21] Amateur Swimming Association (2010). Success is Long Term: Long-Term Athlete Development Related to the Journey through Swimming.

[22] Amateur Swimming Association (2014). The ASA handbook 2014.

[23] Barynina I.I., Vaitsekhovskii S.M. (1992). The aftermath of early sports specialization for highly qualified swimmers. Fitness Sports Rev. Int..

[24] Côté J., Fraser-Thomas J., Crocker P. (2007). Youth involvement in sport. Sport Psychology: A Canadian Perspective.

[25] Sokolovas G., Vilas Boas J.P., Alves F., Marques A. (2006). Analysis of USA swimming's all-time top 100 times. Proceedings of the Xth International Symposium on Biomechanics and Medicine in Swimming.

[26] Allen S.V., Vandenbogaerde T.J., Hopkins W.G. (2014). Career performance trajectories of Olympic swimmers: Benchmarks for talent development. Eur. J. Sport Sci..

[27] Dormehl S.J., Robertson S.J., Williams C.A. (2016). Modelling the progression of male swimmers’ performances through adolescence. Sports.

[28] Mbah A.K., Paothong A. (2015). Shapiro-Francia test compared to other normality test using expected p-value. J. Stat. Comput. Sim..

[29] Witten I.H., Frank E. (2005). Data Mining: Practical Machine Learning Tools and Techniques.

[30] Malina R.M., Bouchard C., Bar-Or O. (2004). Growth, Maturation and Physical Activity.

[31] Whincup P.H., Gilg J.A., Odoki K., Taylor S.J.C., Cook D.G. (2001). Age of menarche in contemporary British teenagers: Survey of girls born between 1982 and 1986. Br. Med. J..

[32] Saavedra J.M., Escalante Y., Rodriguez F.A. (2010). A multivariate analysis of performance in young swimmers. Pediatr. Exerc. Sci..

[33] Wiersma L.D. (2000). Risks and benefits of youth sport specialization: Perspectives and recommendations. Pediatr. Exerc. Sci..

[34] Côté J., Lidor R., Hackfort D. (2009). ISSP position stand: To sample or to specialize? Seven postulates about youth sport activities that lead to continued participation and elite performance. Int. J. Sport Exerc. Psychol..

[35] Cornett A.C., Stager J.M. (2015). Large n: A strategy for improving regional sport performance. Int. J. Sports Physiol. Perform..

[36] Arellano R., Brown P., Cappaert J., Nelson R.C. (1994). Analysis of 50-, 100-, and 200-m freestyle swimmers at the 1992 Olympic games. J. Appl. Biomech..

[37] Ratel S., Duche P., Williams C.A. (2006). Muscle fatigue during high-intensity exercise in children. Sports Med..

[38] Chatterjee S., Laudato M. (1996). An analysis of world record times of men and women in running, skating, and swimming. J. Strength Cond. Res..

